# Supplementary material for: A continuous approach to explain insomnia and subjective-objective sleep discrepancy
Source: Commun Biol. 2025 Mar 12;8:423. doi: 10.1038/s42003-025-07794-6 (PMC11903875; doi:10.1038/s42003-025-07794-6)
Supplement: Supplementary file 1 — Supplementary Material [file 42003_2025_7794_MOESM1_ESM.pdf]

Supplementary Material

Supplementary Methods

Computing hypnodensities using USleep

In addition to our primary way of computing hypnodensities, i.e., using a dedicated classifier to predict sleep stage probabilities with features obtained with catch22, we also utilized USleep (*1*) for comparison. USleep is one of the best-performing and most robust deep learning-based sleep stage classifiers available, having been trained and evaluated on more than 15,000 subjects from 16 different cohorts. Hypnodensity was computed by inputting all the available EEG and EOG channels from a PSG recording. The output was a confidence score for each sleep stage (W, N1, N2, N3, and REM) per 30-second epoch, obtained from each combination of EEG+EOG channels. The output was averaged, obtaining one single probability for each sleep stage per epoch.

Supplementary Figures

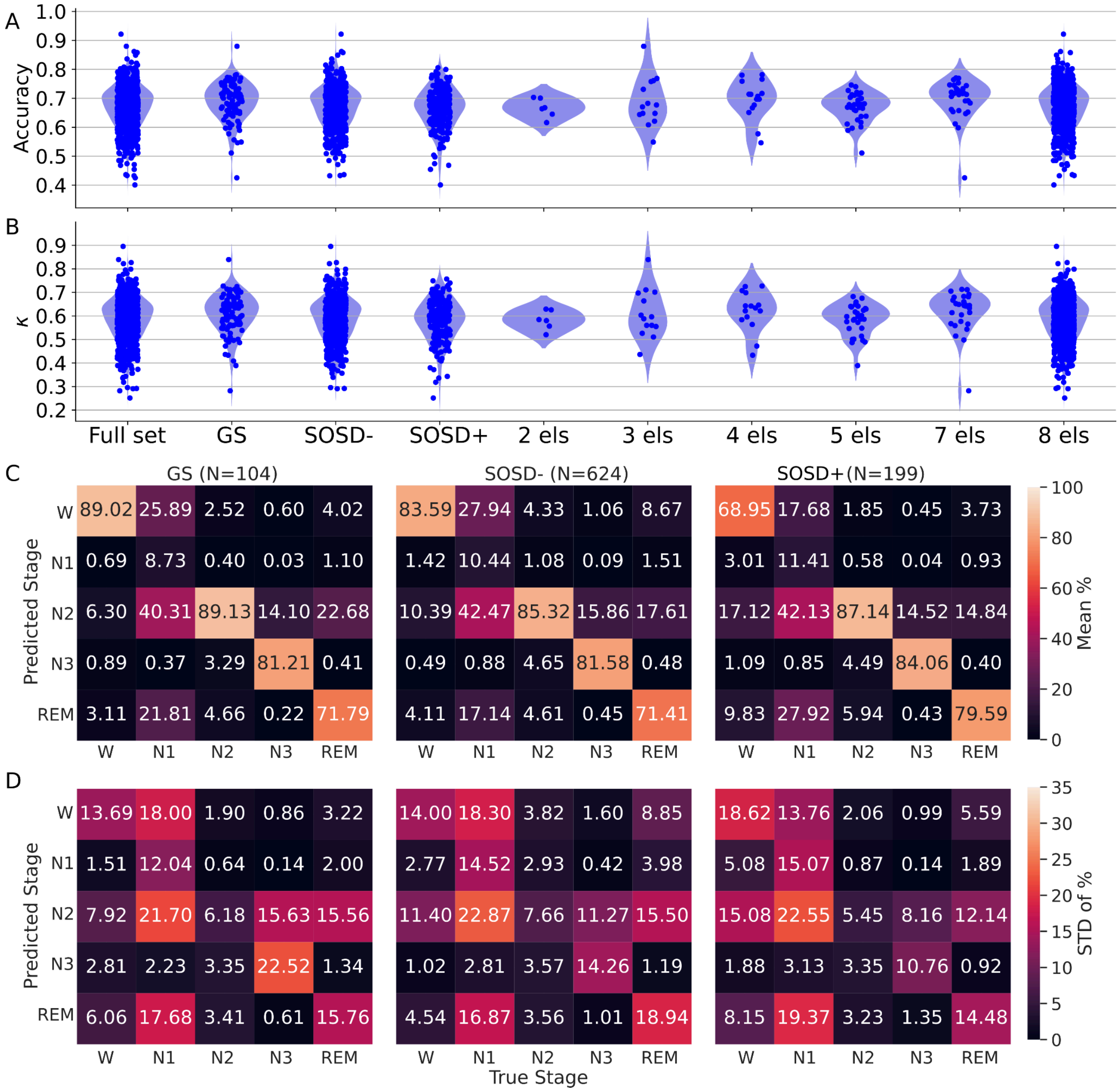

Supplementary Figure 1. **Summary of sleep stage prediction.** **A** and **B** show the balanced accuracy and kappa score, respectively, for the Full dataset (n=927), the specific populations (GS, SOSD- and SOSD+) and for PSG recorded with different numbers of electrodes (e.g. 2 els = 2 electrodes). **C** and **D** are the average and standard deviation, respectively, of the sleep stage prediction confusion matrices.

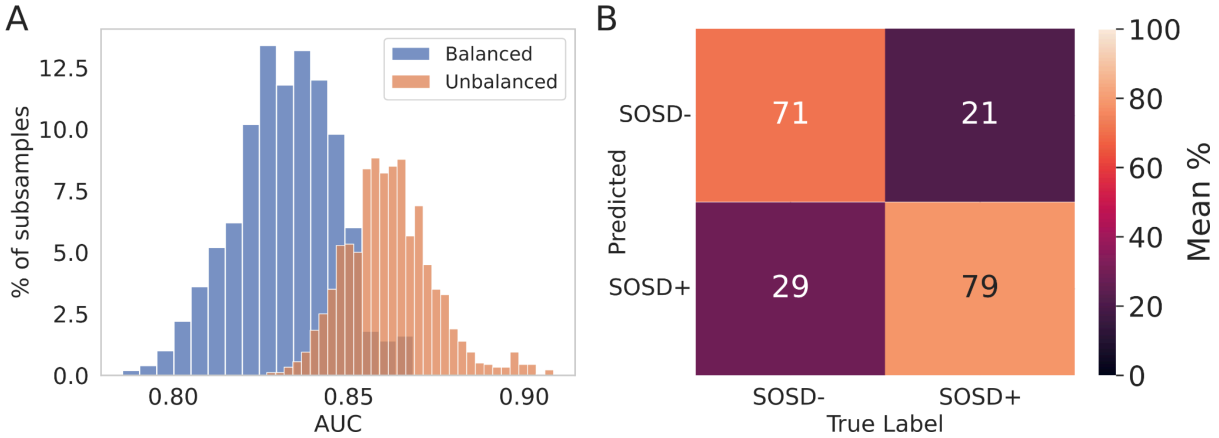

Supplementary Figure 2. **Controlling for sample size, sex, age and BMI. A)** Histogram of the AUC values for the balanced (blue) and unbalanced (red) samples using the best 4 features found for the binary case . **B)** Average confusion matrix for the balanced samples.

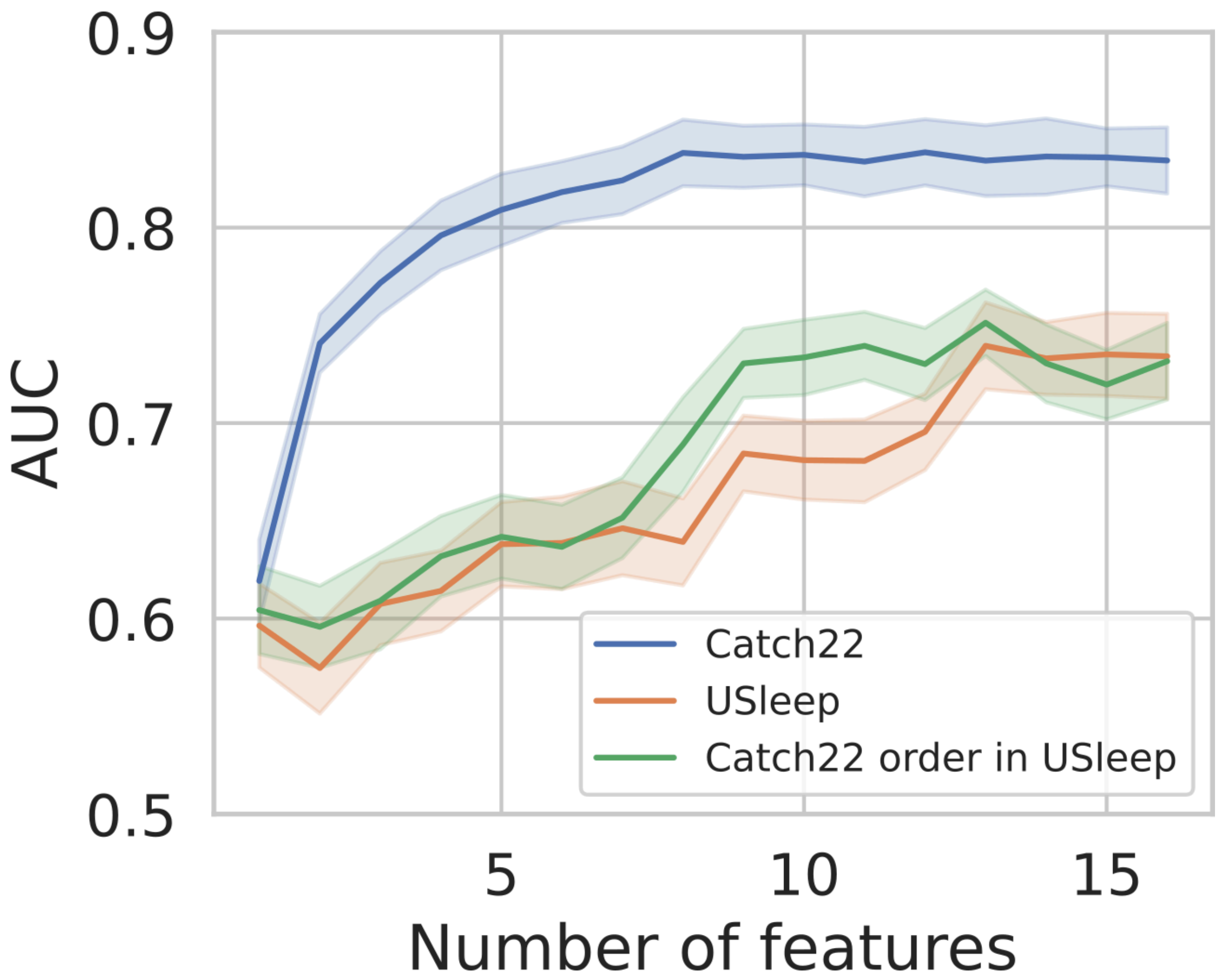

Supplementary Figure 3. **Comparing classifications performance with USleep algorithm.** A subsample of 888 subjects (GS: n=79, SOSD-: n=612, SOSD+: n=197) that satisfied the requisites of the USleep algorithm was used to classify SOSD- vs SOSD+ following the same procedure as in Figure 3. Features were sorted using the MRMR and sequentially included in the classifier. The classification using our hypnodensities inference algorithm, based on catch22 feature extraction (blue), outperformed the classification using hypnodensities extracted with USleep (orange). To further test that the USleep features themselves were less informative than the ones extracted by our algorithm, we sorted them following the ranking obtained for our algorithm (green) and performed the same sequential feature inclusion. None of the USleep based approaches achieved a performance as high as the one obtained with our procedure.

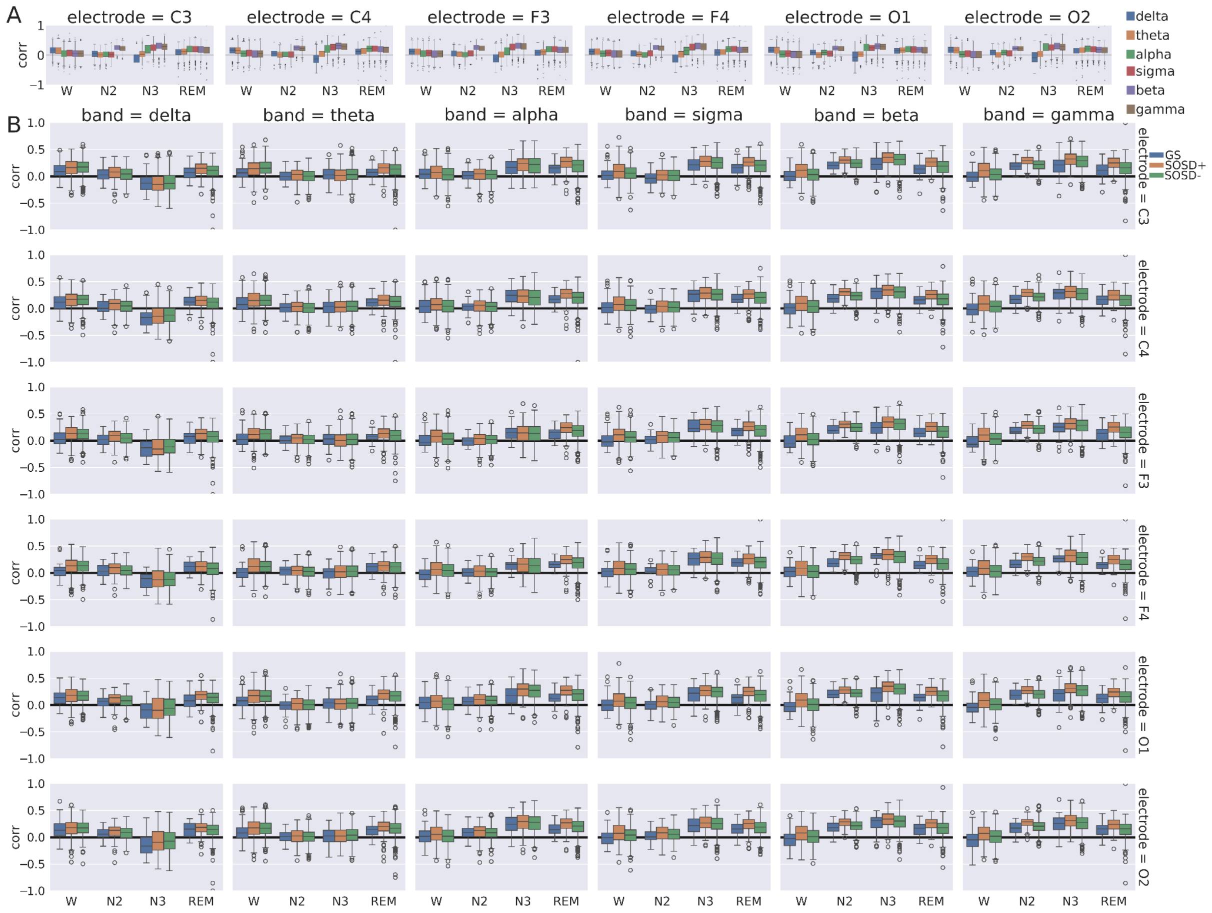

Supplementary Figure 4. **Correlation between entropy and power in EEG frequency bands for all electrode and sleep groups. A)** Correlation between entropy and power in 6 frequency bands for the whole population. Each panel is a different EEG channel. **B)** Same than **A**, but splitting by sleep groups. Rows are different EEG channels.

Supplementary Tables

Supplementary Table 1. **Average Hypnodensities by sleep stage and sleep group.**

	Stage = W					
	GS		SOSD-		SOSD+	
	mean	std	mean	std	mean	std
P(W)	0,753	0,180	0,695	0,164	0,495	0,174
P(N1)	0,031	0,030	0,056	0,051	0,071	0,063
P(N2)	0,125	0,094	0,148	0,116	0,239	0,135
P(N3)	0,024	0,039	0,022	0,023	0,042	0,031
P(REM)	0,067	0,075	0,079	0,053	0,154	0,086
	Stage = N1					
	GS		SOSD-		SOSD+	
P(W)	0,243	0,200	0,261	0,149	0,156	0,098
P(N1)	0,079	0,095	0,104	0,111	0,094	0,099
P(N2)	0,365	0,177	0,396	0,170	0,430	0,161
P(N3)	0,023	0,026	0,037	0,033	0,044	0,054
P(REM)	0,232	0,156	0,185	0,131	0,270	0,155
	Stage = N2					
	GS		SOSD-		SOSD+	
P(W)	0,040	0,034	0,069	0,055	0,034	0,036
P(N1)	0,021	0,026	0,042	0,046	0,030	0,027
P(N2)	0,806	0,092	0,737	0,108	0,771	0,082
P(N3)	0,056	0,043	0,081	0,051	0,084	0,050
P(REM)	0,079	0,039	0,071	0,039	0,081	0,035
	Stage = N3					
	GS		SOSD-		SOSD+	
P(W)	0,021	0,014	0,029	0,020	0,017	0,014
P(N1)	0,006	0,010	0,012	0,014	0,008	0,008
P(N2)	0,174	0,135	0,202	0,109	0,183	0,081
P(N3)	0,742	0,209	0,725	0,148	0,766	0,116
P(REM)	0,018	0,014	0,020	0,014	0,020	0,017
	Stage = REM					
	GS		SOSD-		SOSD+	
P(W)	0,062	0,043	0,109	0,074	0,061	0,057
P(N1)	0,046	0,051	0,048	0,051	0,045	0,040
P(N2)	0,246	0,117	0,203	0,116	0,191	0,089
P(N3)	0,018	0,023	0,023	0,021	0,019	0,018
P(REM)	0,618	0,136	0,605	0,167	0,673	0,135

Supplementary Table 2. **Time spent in each sleep stage (seconds)**

	GS			SOSD-			SOSD+		
**Stage**	**Mean**	**5th Percentile**	**95th Percentile**	**Mean**	**5th Percentile**	**95th Percentile**	**Mean**	**5th Percentile**	**95th Percentile**
W	2003,077	262,500	6009	3800,703	787,500	9210	1240,402	270	2883
N1	800,481	90	2245,500	1172,444	150	3232,500	867,739	117	2103
N2	13496,827	8697	18174	10793,818	6547,500	15472,500	11666,683	7887	15195
N3	4784,423	649,500	8181	4960,879	1807,500	8452,500	5929,749	2817	9672
REM	4741,154	1534,500	7191	4113,930	1380	7042,500	5146,432	2247	7989

Supplementary Table 3. **Statistical comparisons between groups for time spent in each sleep stage**

	P-value	P-value	P-value
Stage	GS vs SOSD-	GS vs SOSD+	SOSD- vs SOSD+
W	<0,001	<0,001	<0,001
N1	<0,001	0,1681	0,0185
N2	<0,001	<0,001	<0,001
N3	0,9032	<0,001	<0,001
REM	0,0298	0,0031	<0,001

P-values were obtained from a Wilcoxon rank sum test with Bonferroni correction for multiple comparisons.

Supplementary Table 4. **Stage normalized frequency**

	GS			SOSD-			SOSD+		
**Normalized Stage Frequency**	**Mean**	**5th Percentile**	**95th Percentile**	**Mean**	**5th Percentile**	**95th Percentile**	**Mean**	**5th Percentile**	**95th Percentile**
W	0,076	0,010	0,229	0,150	0,036	0,337	0,050	0,012	0,120
N1	0,031	0,003	0,084	0,047	0,006	0,133	0,035	0,004	0,080
N2	0,524	0,363	0,683	0,436	0,267	0,610	0,470	0,333	0,592
N3	0,188	0,026	0,332	0,202	0,070	0,350	0,239	0,110	0,387
REM	0,181	0,070	0,267	0,165	0,055	0,267	0,206	0,099	0,301

Supplementary Table 5. **Statistical comparisons between groups for stage normalized frequency**

	P-value	P-value	P-value
Stage	GS vs SOSD-	GS vs SOSD+	SOSD- vs SOSD+
W	<0,001	<0,001	<0,001
N1	<0,001	0,371	0,0182
N2	<0,001	<0,001	<0,001
N3	1	<0,001	<0,001
REM	0,00151	0,201	<0,001

P-values were obtained from a Wilcoxon rank sum test with Bonferroni correction for multiple comparisons.

Supplementary Table 6. **Quantile regression for groups effects on average entropy controlled by stage normalized frequency**

	Entropy					
	**GS vs SOSD-**		**GS vs SOSD+**		**SOSD- vs SOSD+**	
	**Coeff**	**P-value**	**Coeff**	**P-value**	**Coeff**	**P-value**
**W**	-0,0773	**0,0000**	-0,1364	**0,0000**	-0,0590	**0,0000**
**N2**	-0,0324	0,1384	-0,0150	1,0000	0,0175	1,0000
**N3**	-0,0565	**0,0000**	-0,0342	0,0223	0,0223	0,0290
**REM**	-0,0272	0,4087	-0,0214	1,0000	0,0059	1,0000

Quantile regression was performed to test the effect of groups on the median of the average entropy for each stage. The sleep stage normalized frequency was used as a covariate. P-values were corrected by the Bonferroni method.

Supplementary Table 7. **Intrusions (entropy) and stability (D_{KL}) for sleep stages**

	Entropy (bits)						D_{KL} (bits)					
	GS		SOSD-		SOSD+		GS		SOSD-		SOSD+	
Stage	mean	std	mean	std	mean	std	mean	std	mean	std	mean	std
W	0,744	0,342	0,953	0,302	1,273	0,240	0,297	0,193	0,351	0,166	0,423	0,235
N1	1,166	0,235	1,254	0,230	1,249	0,226	0,471	0,385	0,425	0,329	0,447	0,342
N2	0,692	0,247	0,912	0,292	0,815	0,230	0,435	0,114	0,420	0,135	0,397	0,110
N3	0,635	0,210	0,773	0,217	0,683	0,204	0,372	0,160	0,353	0,160	0,355	0,135
REM	0,996	0,190	1,084	0,259	1,002	0,220	0,518	0,205	0,470	0,208	0,437	0,166

Supplementary Table 8. **Quantile regression for groups effects on average Kullback-Leibler controlled by stage normalized frequency**

	DKL					
	**GS vs SOSD-**		**GS vs SOSD+**		**SOSD- vs SOSD+**	
	**Coeff**	**P-value**	**Coeff**	**P-value**	**Coeff**	**P-value**
**W**	0,1675	**0,0000**	0,0792	**0,0001**	-0,0883	**0,0000**
**N2**	0,0155	0,0161	0,0194	0,0024	0,0039	1,0000
**N3**	0,0299	**0,0000**	0,0284	**0,0000**	-0,0015	1,0000
**REM**	0,0419	**0,0000**	0,0570	**0,0000**	0,0152	0,1219

Quantile regression was performed to test the effect of groups on the median of the average Kullback-Leibler divergence for each stage. The sleep stage normalized frequency was used as a covariate. P-values were corrected by the Bonferroni method.

Supplementary Table 9. **Pairwise group comparisons on subject characteristics**

	P-values*		
	GS vs SOSD+	SOSD+ vs SOSD-	GS vs SOSD-
Age	0,182	8,925E-06	9,675E-10
Gender	2,55E-11	1	3,765E-12
BMI	1	1	1
ESS	1	1	1
Smoking	1	1	1
Depression	0,941	1	0,398
Anxiety	1	1	1
Cardiovascular	1	0,255	1
Pulmonaire	1	1	1
Gastro	1	1	1
Infectious disease	1	1	1
Metabolic	1	1	1
Cancer	1	1	1
Covid	1	1	1
Epilepsy	1	1	1
Pain	1	1	1
Other Comorbidity	1	1	1
Antidepressant	0,736	1	0,609
Antihistamine	1	1	1
Antipsychotic	1	1	1
Benzodiazepine	0,483	1	0,377
Melatonin	1	1	1
Z Drugs	1	1	1
Opioid	1	1	1
Other Medication	0,0343	1	0,0117

The table shows the group comparisons for values presented in Table 1. We used a t-test for continuous variables and a chi-square for categorical variables. *Bonferroni corrected for the total number of comparisons performed with each test.

Supplementary References

1. Perslev, M. *et al.* U-Sleep: resilient high-frequency sleep staging. *Npj Digit. Med.* **4**, 1–12 (2021).
