# Supplementary material for: A continuous approach to explain insomnia and subjective-objective sleep discrepancy
Source: Commun Biol. 2025 Mar 12;8:423. doi: 10.1038/s42003-025-07794-6 (PMC11903875; doi:10.1038/s42003-025-07794-6)
Supplement: Supplementary file 2 — Reporting Summary [file 42003_2025_7794_MOESM2_ESM.pdf]

nature portfolio

Corresponding author(s): Thomas Andrillon

Last updated by author(s): Feb 18, 2025

Reporting Summary

Nature Portfolio wishes to improve the reproducibility of the work that we publish. This form provides structure for consistency and transparency in reporting. For further information on Nature Portfolio policies, see our Editorial Policies and the Editorial Policy Checklist.

Statistics

For all statistical analyses, confirm that the following items are present in the figure legend, table legend, main text, or Methods section.

n/a	Confirmed	
☐	☒	The exact sample size (n) for each experimental group/condition, given as a discrete number and unit of measurement
☐	☒	A statement on whether measurements were taken from distinct samples or whether the same sample was measured repeatedly
☐	☒	The statistical test(s) used AND whether they are one- or two-sided<br>*Only common tests should be described solely by name; describe more complex techniques in the Methods section.*
☐	☒	A description of all covariates tested
☐	☒	A description of any assumptions or corrections, such as tests of normality and adjustment for multiple comparisons
☐	☒	A full description of the statistical parameters including central tendency (e.g. means) or other basic estimates (e.g. regression coefficient) AND variation (e.g. standard deviation) or associated estimates of uncertainty (e.g. confidence intervals)
☐	☒	For null hypothesis testing, the test statistic (e.g. F, t, r) with confidence intervals, effect sizes, degrees of freedom and P value noted<br>*Give P values as exact values whenever suitable.*
☒	☐	For Bayesian analysis, information on the choice of priors and Markov chain Monte Carlo settings
☒	☐	For hierarchical and complex designs, identification of the appropriate level for tests and full reporting of outcomes
☒	☐	Estimates of effect sizes (e.g. Cohen's d, Pearson's r), indicating how they were calculated

Our web collection on statistics for biologists contains articles on many of the points above.

Software and code

Policy information about availability of computer code

Data collection	The study employed multiple PSG devices, including NOX-A1 (Nox Medical, N=820), ACTIWAVE (CamNTech Ltd, N=83), MORPHEUS (Micromed S.p.A., N=13), and SOMNOLOGICA (Medcare, N=11). All devices utilized in the study were validated and routinely employed in the sleep center and/or IRBA laboratory settings.
Data analysis	All data was analyzed using python 3.12<br>Computational codes to extract hypnodensities and compute intrusions and instability are available in https://zenodo.org/doi/10.5281/zenodo.13143110.

For manuscripts utilizing custom algorithms or software that are central to the research but not yet described in published literature, software must be made available to editors and reviewers. We strongly encourage code deposition in a community repository (e.g. GitHub). See the Nature Portfolio guidelines for submitting code & software for further information.

Data

Policy information about availability of data

All manuscripts must include a data availability statement. This statement should provide the following information, where applicable:

- Accession codes, unique identifiers, or web links for publicly available datasets
- A description of any restrictions on data availability
- For clinical datasets or third party data, please ensure that the statement adheres to our policy

Due to protection of personal privacy, the clinical database used in this work cannot be publicly available. However, the data can be provided by DL pending scientific review and a completed material transfer agreement. Requests for the PSG recordings and associate metadata should be submitted to damien.leger@aphp.fr.

Human research participants

Policy information about studies involving human research participants and Sex and Gender in Research.

Reporting on sex and gender	Only sex was reported as gender was not asked to patients nor participants.
Population characteristics	All population characteristics is reported on Table 1.
Recruitment	The recruitment strategy differed between patients with chronic insomnia and good sleepers. PSG recordings from patients were obtained as part of their clinical care whereas PSG recordings from good sleepers were obtained as part of their participation in research protocols (see details below). In both cases, participants provided written consent and approved the re-use of their data for research purposes. Chronic insomnia was diagnosed following the International Classification of Sleep Disorders, Third Edition (ICSD-3) 16, and defined as a complaint of persistent difficulties in sleep initiation, maintenance, or early morning awakenings for a minimum of three nights per week over a span of at least three months 15. Patients were screened for obstructive sleep apnea (OSA) or periodic limb movement disorder (PLMD) by analysing PSG recordings and following existing guidelines (ICSD-3)16. All individuals with OSA and PLS were excluded from our analyses.<br>The initial baseline night from prior protocols was consistently selected for analysis, conducted by the VIFASOM team. Control subjects underwent rigorous screening to exclude confounding factors such as sex, age, and comorbidities. Exclusions included individuals with OSA, PLMD, or total sleep time outside the range of 4 to 8 hours. In addition, subjects with chronic unbalanced pathologies, psychotropic treatments or those that could affect sleep were excluded from the protocols. Shift and night workers, people exposed to jet-lag or irregular sleep schedules (based on a 2-week sleep diary) were also excluded.
Ethics oversight	Comité de Protection des personnes (CPP) Ouest IV, Nantes, France)

Note that full information on the approval of the study protocol must also be provided in the manuscript.

Field-specific reporting

Please select the one below that is the best fit for your research. If you are not sure, read the appropriate sections before making your selection.

☒ Life sciences ☐ Behavioural & social sciences ☐ Ecological, evolutionary & environmental sciences

For a reference copy of the document with all sections, see nature.com/documents/nr-reporting-summary-flat.pdf

Life sciences study design

All studies must disclose on these points even when the disclosure is negative.

Sample size	This is a retrospective study, so sample size was as big as possible based on the database availablility.
Data exclusions	The total number of available recordings was 1042. However, 115 recordings were discarded because of noisy data or because the hypnogram was predicted with a balanced accuracy lower than 0.4
Replication	As a retrospective analysis, there was no replication, as calling all the patients and subjects back to the clinic involves a big effort for them and for the hospital resources.
Randomization	This is a retrospective study, no randomization was performed
Blinding	This is a retrospective study, no blinding was performed

March 2021

Reporting for specific materials, systems and methods

We require information from authors about some types of materials, experimental systems and methods used in many studies. Here, indicate whether each material, system or method listed is relevant to your study. If you are not sure if a list item applies to your research, read the appropriate section before selecting a response.

Materials & experimental systems

n/a	Involved in the study
☒	☐ Antibodies
☒	☐ Eukaryotic cell lines
☒	☐ Palaeontology and archaeology
☒	☐ Animals and other organisms
☒	☐ Clinical data
☒	☐ Dual use research of concern

Methods

n/a	Involved in the study
☒	☐ ChIP-seq
☒	☐ Flow cytometry
☒	☐ MRI-based neuroimaging
